# Remarks on a Species of Intermittent Tetanus; with Cases

**Published:** 1831-09

**Authors:** M. Dance


### INTERMITTENT TETANUS.

### *Remarks on a Species of Intermittent Tetanus; with Cases.* By M. DANCE.*

THE title of intermittent tetanus is given by M. DANCE to a disease which he has not found described by any writer, unless we identify it with that variety of malignant intermittent fever, indicated by authors by the term *tetanic.* He points out, however, various distinctions which exist between these diseases: the former proceeds by more or less regular paroxysms; it terminates spontaneously and favorably, notwithstanding its strong resemblance to tetanus. The following cases are related.

CASE I. A female servant, twenty-five years of age, of a sanguine temperament, was admitted into the hospital, October 5th, 1824. She had menstruated from the age of fourteen, and had always enjoyed perfect health, and had also passed through pregnancy and labour without any unfavorable circumstances. About

* Archives Génerales, Juin 1831.

four months ago, and a short time after delivery, she was attacked, without any evident cause, by a sort of fit, without loss of sense, which was characterized by numbness in different parts of the body, and followed by stiffness in the limbs, similar to that which occurs in the most painful kinds of cramp. At subsequent periods similar attacks occurred, at irregular intervals; each paroxysm lasting three or four hours. Latterly, the attacks had been more frequent and of longer duration, and at length the patient was taken into the hospital in the following state: The forearm was half bent; hands closed; the inferior extremities extended, and also the feet. All these parts were so stiff that considerable force was required to alter their position, which was immediately resumed when they were left to themselves. Violent startings of the limbs frequently occurred, attended with so much pain as to cause the patient to utter loud cries. The skin was warm, and covered with sweat; face flushed and animated; respiration accelerated; pulse hard and quick; tongue of a natural appearance; intellectual faculties undisturbed. Under these circumstances, it was deemed proper, considering the intensity of the febrile symptoms, to have recourse to a free bleeding, although the patient stated that no benefit had before been derived from venesection: she thought that it had rather increased the symptoms; and, indeed, after she had now been bled, the stiffness and pain of the limbs were augmented, but the following night she was more free from pain, and the limbs were somewhat relaxed.

On the morning of the 6th, another attack came on, similar to that of the preceding day, and without any previous shivering; the forearms and fingers were firmly bent; extension of the thighs, legs, and feet; the muscles of the affected parts were contracted, and hard: they felt like tense cords, and were occasionally thrown into more violent contractions. During this aggravation of the symptoms, the patient screamed out from pain; the skin was covered with perspiration, and the pulse was as full and quick as it had been the evening before. A warm bath was ordered; poultices, sprinkled with laudanum, were applied to the limbs; an antispasmodic mixture. She was kept in the bath for three hours, and was much relieved; the limbs were almost entirely relaxed, and she had a calm night.

7th. At six o'clock in the morning, the pain and stiffness of the limbs came on with the same violence as before; and, as usual, the superior extremities were in a state of flexion, the inferior extended, and at every instant the patient cried out from the excessive anguish in her shoulders, arms, and calves of the legs. She was again relieved by a prolonged use of the bath.

8th. Another attack in the morning, precisely the same as before, but not relieved by the bath, and continuing until the 9th, when the patient was nearly in the same state; the stiffness of the limbs and the febrile symptoms having somewhat abated. In the evening, a slight menstrual discharge occurred, and twelve leeches were applied to the thighs.

10th. No attack. Limbs flexible, but numb, and occasionally cramped. This amendment continued until the 15th, when a fresh attack came on, but much milder than before: it continued through the 16th, 17th, and 18th, and was accompanied by a sensation of constriction in the throat, as is observed in hysteria. Warm bath repeated; assafœtida enema.

From this time the stiffness and pain of the limbs entirely ceased, and the patient soon quitted the hospital in perfect health.

This was the first case M. Dance saw of this singular disease. The principal difficulty was to determine the nature of the malady. Was it to be considered a case of simple cramp, muscular rheumatism, tetanus, an anomalous case of hysteria, an idiopathic affection of the muscles, or some lesion of the spinal marrow, or of the brachial and lumbar nerves. Some of the phenomena resembled each of these affections, but it is evident that collectively they would not apply to either. In cramp there is no fever, and the symptoms are of short duration; in rheumatism, the symptoms do not come on in paroxysms similar to those above described; the progress of tetanus is very different; in hysteria, however various the symptoms may be, fever is never present.

Case II. A steel polisher, seventeen years old, and very powerful for his age, of good health in general, had been subject for two years to attacks, during which his limbs became suddenly so stiff that he was prevented from executing any movement. At first these attacks lasted but a short time, and ceased spontaneously; they were afterwards longer, disappeared more gradually, and left the limbs benumbed. They came on at irregular intervals. On the 19th of March, 1826, a paroxysm of the same kind occurred, which lasted only one hour: the 21st, he was admitted into the Hôtel Dieu.

At the time of his admission, there was nothing particular in his appearance, and the history he gave of his previous attacks was considered obscure, and even of doubtful accuracy; but about ten o'clock he was seized with a stiffness of his limbs, which continued to increase, and in the evening he was in the following state: The fore-arms were rigidly bent upon the upper arms; fingers in close contact, and bent, as were also the wrists; the inferior extremities rigidly extended; all the parts affected were painful; he was restless, and continually moving his body in various positions; face deeply coloured; abundant perspiration; great thirst; pulse full, hard, and rapid. In this state he remained throughout the night, constantly groaning and complaining.

22d morning. Face still more deeply coloured; agitation increased; occasionally sudden and painful startings in the contracted limbs; the labial muscles being also affected, and hence his speech was somewhat impeded; intellect clear. He was free from pain in every other part except the limbs. He was very anxious to be bled immediately, and his wish was complied with: about sixteen ounces of blood were taken away; it was not buffed. An antispasmodic mixture was ordered. No relief was obtained from the bleeding.

In the evening, the same state of flexion and stiffness of the forearms, fingers, and wrists; the inferior extremities remained extended, and the feet were rigidly stretched out; painful and violent startings of the muscles of these parts still occurring from time to time. The muscles felt very hard and tense, and, when the hand was passed over them, a convulsive vibration was felt. All these phenomena were more strongly marked on the left, than on the right side. The muscles which form the margins of the axillæ, as well as the masseters, were also hard and tense: the former kept the arms fixed to the trunk; the latter impeded the opening of the mouth, without, however, rendering it impossible. The other muscles of the trunk did not participate in the stiffness, and the patient could turn himself, sit down, or raise himself at will; he could even get out of bed, and stand up for a few moments, when relaxation of the inferior extremities took place, but he could not extend the fore-arms or the fingers. There was no pain along the spine; fever unabated; perspiration copious. He was ordered to be put into a warm bath, but he could only remain in about ten minutes, on account of the distress he felt from the position; the pains were also much increased. Limbs to be rubbed with laudanum; an enema with ten drops of laudanum.

23d. Passed a better night. Less stiffness of the limbs; can slightly extend the fore-arms and fingers; less fever; great appetite. Bath; enema with assafœtida ʒi.; antispasmodic mixture.

24th. Has had a good night, and better in every respect. He rose this morning, and can walk with tolerable facility, but he drags the left foot a little. Can extend the fore-arms and fingers; no contraction of the jaw; pulse natural. Remedies continued.

From this time the power and mobility of the limbs gradually returned, but it was not until the 18th of the ensuing month that he could use them as freely as before: until that time he occasionally felt a slight numbness, and passing stiffness, especially in the limbs of the left side.

M. Dance saw this patient three years afterwards, and he had not had any relapse.

This case differs only from the preceding in the greater degree of violence of the symptoms. In both, the disease commenced by mild and distant attacks, which did not appear at regular intervals, and continued to increase in severity and frequency, until a paroxysm, more violent and prolonged than the former, appeared to terminate the disease.

The attacks were characterized by numbness and stiffness, and even by violent and continued contractions of the same order of muscles, followed by painful startings, which coincided with the occasional aggravation of the tetanic contractions. The muscles thus affected belonged to the upper and lower extremities, and hence arose the rigid flexion of the forearms and fingers, and an equally powerful extension of the legs and feet. In one case the disease extended to the masseter muscles, and a kind of trismus was produced. To these phenomena were added intense fever, great agitation, general heat, redness of the face, and abundant perspiration. The resemblance between the symptoms of these two cases proves the identity of the disease; and although it may be impossible to assign to it any established character, it must at least be inferred, from this resemblance, that the disease, whatever it might be, is not one of those rare cases which constitutes an undiscoverable pathological anomaly, since it appeared in the same form in two persons, of different ages and different sexes. In the following instances also the disease preserved the same characters.

Case III. A goatkeeper, forty-six years of age, of ordinary stature, rather thin, and generally of good health, had just been confined six months in prison for some act of violence, when he was attacked with sudden stiffness and numbness of the limbs, which prevented him from assisting himself. He remained in this state fifteen days, with occasional diminution and aggravation of the symptoms. On the seventeenth day, February 2d, 1826, he was seized with more violent stiffness, which produced an involuntary flexion of the fore-arms and fingers; the inferior extremities were benumbed. This attack lasted four or five hours, and was accompanied by heat of the skin, redness of the face, and great perspiration. These symptoms were followed by a general sensation of soreness, as if the limbs had been violently bruised.

3d. Patient felt tolerably well.

4th. Admitted into the Hôtel Dieu, where he passed the day without any complaint: he appeared, indeed, in so good a state of health, that it was presumed he wanted rather the comforts of a hospital than medical aid; but, about the middle of the following night, he was awakened by pains, numbness, and stiffness of the muscles of the extremities, which continued to increase.

When he was visited at seven o'clock the next morning, he was lying on his back, and as immoveable as a statue: the fore-arms were so rigidly bent that great force was required to extend them; hands firmly closed; the thumb of the left hand was covered by the fingers, but that of the right hand was in contact, at its extremity, with the index finger, as when a pen is held in writing. The inferior extremities were extended, and were nearly as rigid as the upper limbs: the muscles of the thigh and calf were as hard and tense as during the most violent cramp. The anterior abdominal muscles presented an analogous rigidity; those of the thorax and neck were not affected; the masseter muscles were contracted, and kept the jaws so closely in contact that much difficulty was experienced in opening the mouth; the labial muscles were slightly affected, and speech was a little impeded. All the muscles thus affected were occasionally attacked with violent and painful startings, which the patient attempted in vain to resist. Pulse quick and hard; respiration irregular and distressed; intellectual faculties unclouded. A few moments after this examination, all the symptoms mentioned became still more severe: the muscular tension was greater, respiration quicker and more distressed, pulse more rapid. The patient was now afflicted by the most painful convulsive startings, and cried and groaned incessantly. At about nine o'clock, the face, which had hitherto retained its ordinary colour, was flushed and animated in appearance: an abundant perspiration broke out, and was accompanied by a slight relaxation of the affected muscles; the pulse, at the same time, was large, full, and undulating. The patient ceased to complain.

At ten o'clock, the sweating had increased, and formed a humid atmosphere of vapour around the patient: the pulse lost its frequency, the tension of the muscles gradually diminished; but still the upper extremities remained bent.

At noon, the perspiration was gradually ceasing, and the limbs began to regain their power of motion.

In the evening, but slight traces of the attack remained. No particular treatment was had recourse to.

On the following day, the patient said he was well: he was free from fever, had a good appetite, and could make use of all his limbs, in which he experienced only a little uneasiness; the tension of the abdominal and masseter muscles had entirely ceased.

This man was kept in the hospital for some months, as an assistant in the infirmary, and remained in perfect health.

When we consider, says M. Dance, the progress of this attack, it might be regarded as a paroxysm of intermittent fever, the three stages of which were marked: the first, not by shivering, but by numbness and stiffness of the muscles, which gradually increased, and were accompanied by fever, which became more and more intense, (second stage,) until a general relaxation occurred, as the consequence of abundant perspiration, (third stage.) When we reflect upon the extraordinary form of the symptoms, we might also presume that the fever belonged to that class of malignant intermittents to which the name of *tetanic* has been given. What, indeed, did the phenomena require, which occurred during the paroxysm, to constitute a true case of tetanus? There was violent trismus, permanent contraction of the abdominal muscles, and a general and involuntary stiffness of the muscles of the extremities, similar to that which existed in the preceding cases. But the paroxysms did not follow the regular progress which they do in legitimate intermittent fevers: there was no shivering at their commencement, and they suddenly ceased without being opposed by any special therapeutic means; whilst malignant intermittent fevers quickly endanger the life of the patient, unless recourse be had to bark. These considerations induce M. Dance to believe that such was not the real nature of the malady. In the following example the attack was still more severe.

Case IV. A printer, fifty-two years old, of a sanguine temperament and robust frame, had experienced two attacks, within five months, of numbness and stiffness of the limbs. In February 1830, he caught a violent cold, at the termination of which he had another and more violent attack, similar to the former. Another occurred on the 4th March: it commenced about seven o'clock in the morning, by a general creeping sensation, which was quickly followed by fever, heat, and rigidity of all the limbs. This attack terminated, at about eleven o'clock, by abundant perspiration.

On the following day, the same symptoms returned in the same order, and at the same hour, but with greater severity, and did not cease till noon. In the evening, the patient was admitted into the Hôtel Dieu, free from fever or any important symptom. He passed a perfectly good night; but on the third day, at seven o'clock in the morning, the medical attendants witnessed the following attack: It commenced by general torpor, transient horripilations, and soon stiffness of the limbs, which continued to increase until the lower extremities became powerfully extended, and were almost inflexible; the upper extremities also were thrown into a state of complete flexion, together with the fingers, and became equally rigid. Gradually the vertebral muscles were affected with the same rigidity, and the trunk was slightly curved backwards: the cervical region alone was under voluntary control. At the same time, the skin was very hot; the pulse too quick to be numbered; respiration much accelerated; intense flushing of the face; urgent thirst; speech impeded, from the difficulty experienced in projecting and moving the tongue, the muscles of which appeared to be affected in a similar manner to those of the limbs. He could open his mouth with facility. Violent and painful startings and convulsions occurred frequently in the contracted parts, and caused the patient to groan and scream.

At half-past eight o'clock, the symptoms had arrived at their highest degree of intensity: an abundant sweat then broke out, which bathed the whole surface of the body. The rapidity of the pulse now gradually diminished, as well as the flush upon the face. At ten o'clock, the upper extremities were relaxed, and the fingers were extended. At eleven o'clock, the trunk and lower extremities were also relaxed, the pulse became quiet, and the paroxysm ceased. The patient stated it to be the most severe he had suffered. At five in the evening, he was perfectly calm; there was not the least stiffness in any of the muscles. He complained of feeling as if he had been bruised. The urine, after the attack, did not shew any deposit.

On the fourth day another paroxysm was expected, but the patient complained only of slight uneasiness in his limbs: they were not at all rigid, nor were there any symptoms of fever.

At the end of three weeks, the patient quitted the hospital in perfect health, without having had any return of the disease. In this case it was not deemed necessary to have recourse to any particular treatment.

The intermittent nature of the disease, and its returns by periodical attacks, were more strongly marked in the last than in the preceding instances. Three attacks, of a quotidian type, and each of increased severity, took place after slight preliminary symptoms had occurred for several days. Each of these attacks was characterized by tetanic phenomena, which, in the last, affected not only the muscular system of the limbs, but that also of the tongue and the posterior part of the trunk, from which resulted impeded speech and true opisthotonos. All these phenomena disappeared with the termination of the paroxysm by an abundant sweat. It cannot, therefore, be doubted that this disease belongs to the class of intermittent fevers; but the term *pernicious* cannot be added, notwithstanding the formidable symptoms which attended it, for it ceased spontaneously, and left the patient in as good a state of health as before the attack.

The following is a short *résumé* of the above cases, with the points in which they resembled each other, and the chief differences which existed between them.

All the patients had first experienced, for two, three, or even four months, slight preliminary attacks of the disease which ultimately brought them to the hospital. These attacks were feeble and of short duration, occurring at irregular intervals, and consisting of slight and transient stiffness in the limbs, which ceased spontaneously, leaving the limbs weak and torpid. Afterwards the attacks became more severe, and assumed a more regular type: quotidian in case IV., tertian in case III., and simply remittent in cases I. and II.; being also accompanied by febrile symptoms analogous to those of an intermittent, with the exception of the shivering stage, which either did not exist at all or was imperfectly developed. At the commencement of the attack, the patients complained of stiffness and numbness in the limbs; but very shortly the muscles became painfully tense, and then very rigid, so that no voluntary motion could be performed, and it was difficult even to move the parts with force. In this state the forearms and fingers were violently bent; the inferior extremities, on the contrary, were extended; and these positions were retained during the whole course of the paroxysm. To these phenomena, which were common to all the four cases related, were added others of the same nature, which gave to the disease the greatest resemblance to tetanus. Thus, in case II., the jaws were in a state of trismus, from contraction of the masseter muscles; in case III. the abdominal muscles were affected in a similar manner; in case IV. the vertebral muscles became rigid, and hence arose opisthotonos. The painful startings and spasms of the contracted muscles, which took place during the height of the paroxysm, complete the resemblance between this disease and tetanus. At the same time, the face was highly flushed, the pulse and respiration were more and more accelerated; the patients laboured under great anxiety, and cried out and moaned from pain. Lastly, all the phenomena continued to increase for some hours, and arrived at the highest degree of intensity, when a most copious perspiration gradually produced a general relaxation, which was speedily followed by the termination of the attack, (cases III. and IV.) or by a simple remission, (cases I. and II.,) to which succeeded a fresh paroxysm, after an uncertain lapse of time. In the second case, the paroxysm appeared to be prolonged for several days; in the third case, it terminated in eight or ten hours. It is also remarkable that the disease, so formidable in its appearance, ended spontaneously, after two, three, or four attacks, without the employment of any particular treatment. In cases I. and II. bleeding was had recourse to, without avail: in the former, the warm bath appeared of service.

M. Dance has not yet made up his mind as to the precise nature of the disease: it has a certain affinity with cramp, some rheumatic affections, tetanus, and intermittent fevers, but does not present the *ensemble* of the symptoms which are proper to either of these maladies. If we regard the phenomena, it appears most like tetanus; its progress more resembles intermittent or remittent fevers. It may, perhaps, be considered a *tetanic intermittent;* and, from the severity of its symptoms, it might be classed among the *fièvres pernicieuses:* but its spontaneous and favorable termination prevents it from being ranged in that order of febrile diseases. M. Dance confesses that he has, as yet, seen too little of this affection to justify him in speaking positively respecting its nature. The facts he has recorded are, however, extremely interesting, and will no doubt fix the attention of practitioners to the subject, and lead to additional investigation and evidence.

---

